# 

**Published:** 1980

**Authors:** 


NATIONAL

LIBRARY

JAN 19 1982

OF

MEDICINE

### BRISTOL MEDICO-CHIRURGICAL JOURNAL

JANUARY/APRIL 1980
VOLUME 95 (i/ii)
NUMBER 353/354

